# A Graphene-PEDOT:PSS Modified Paper-Based Aptasensor for Electrochemical Impedance Spectroscopy Detection of Tumor Marker

**DOI:** 10.3390/s20051372

**Published:** 2020-03-02

**Authors:** Yi-Kuang Yen, Chen-Hsiang Chao, Ya-Shin Yeh

**Affiliations:** 1 Department of Mechanical Engineering, National Taipei University of Technology, Taipei 106, Taiwan; t107568006@ntut.edu.tw; 2 Taiwan Semiconductor Manufacturing Company, Hsinchu 30078, Taiwan; t106408088@ntut.edu.tw

**Keywords:** electrochemical impedance spectroscopy, carcinoembryonic antigen, paper-based device, graphene, conductive polymer, aptamer

## Abstract

A graphene and poly (3,4-ethylenedioxythiophene):poly(styrenesulfonate) (PEDOT:PSS) modified conductive paper-based electrochemical impedance spectroscopy (EIS) aptasensor has been successfully fabricated by a simple and continuous coating process. A graphene/PEDOT:PSS modified paper electrode forms the nanocomposite providing a conductive and sensitive substrate for further aptamer functionalization of the biosensor. This low-cost paper-based aptasensor exhibits its sensitivity to carcinoembryonic antigens (CEA) in standard buffer solutions and human serum samples in a linear range of 0.77–14 ng·mL^−1^. The limit of detection (LOD) is found to be 0.45 ng·mL^−1^ and 1.06 ng·mL^−1^ for CEA in both samples, separately. This aptamer-based sensing device was also evaluated and received a good correlation with the immunoassay detection method. The proposed paper-based aptasensor has demonstrated its potential as a rapid simple point-of-care analytical platform for early cancer diagnosis in less developed areas where manufacturing facilities, analytical instruments, and trained specialists are limited.

## 1. Introduction

Developing sensitive and reliable point-of-care (POC) devices for early cancer screening, diagnosis, and treatment monitoring is an important task in both the developing and developed world [1,2]. Promising interdisciplinary research grows on this context because POC analytical platforms can reduce cost, decrease the sample amount, achieve an on-site diagnosis and mitigate patient stress [3,4]. Additionally, with the discovery of tumor biomarkers, such as alpha-fetoprotein, prostate specific antigen (PSA) and carcinoembryonic antigen (CEA), the feasibility of analysis in oncology has been enhanced [5]. CEA is one of the most widely used tumor markers associated with colorectal cancer, pancreatic cancer, gastric cancer, and lung cancer [6]. Generally, the normal serum level of CEA in humans should be less than 5 ng/mL which is the cutoff value for the indication of cancer [7,8]. In addition, changes in serum CEA levels in patients with colorectal cancer can be used to detect early recurrence after surgery [7]. It also has been reported that elevated serum CEA levels might play an important role in predicting a poor prognosis for pancreatic cancer patients [9].

The clinical detection of CEA is mainly based on immunoassays. For example, chemical luminescence microparticle immunoassay (CMIA) and electrochemiluminescence immunoassay (ECLIA) are the two most commonly used immunoassays in hospitals [10]. The advantages of these methods are automatic batch inspection, simple operation, and wide detection intervals, but the disadvantage is that the equipment needed is expensive and cumbersome. In addition to conventional immunoassays, a Love Wave surface acoustic wave (SAW) sensor has been reported for the automatic and online detection of CEA in exhaled breath condensate [11]. Electrochemical immunosensors have recently been of interest to scientists due to their captivating properties, which include high sensitivity, fast response, short diagnostic time, and miniaturization [12]. For example, a new electrochemical immunosensor based on an AuNPs/PEDOT/GR composite was fabricated for enhancing the detection sensitivity of CEA in real human serum [13]. Furthermore, a conducting paper-based electrochemical immunosensor was developed for CEA sensing by progressively creating a poly (3,4-ethylenedioxythiophene):poly(styrenesulfonate) (PEDOT:PSS) film directly over the paper substrate [14]. This approach demonstrated the advantageous properties of a paper-based sensor including flexibility, lightweight, low cost, easy fabrication, biocompatibility, and biodegradability, which satisfied the demand for developing disposable POC devices for areas with a shortage of medical resources. Besides electrochemical immunosensors, a microfluidic paper-based electrochemical aptasensor was fabricated through wax printing and screen printing for simultaneous detection of CEA and neuron-specific enolase (NSE) in a clinical sample [15]. This method provided a great sensitivity of detection and a possible low-cost platform. However, the patterning processes for the devices and the synthesis of nanocomposites for sensing electrodes depend on customized equipment and well-trained personnel. These requirements might be difficult to satisfy in areas with limited resources, or remote or rural communities, where the demand for analytical devices is clearly evident [16].

In this work, a paper-based electrochemical aptasensor for CEA detection was developed using graphene ink and PEDOT:PSS progressively modified on paper substrate to form a conductive composite paper electrode. Graphene has excellent characteristics including ideal mechanical strength, good electrical conductivity and a high surface-to-volume ratio which makes surface transporting electrons highly sensitive to adsorbed molecules [17,18]. It also has been reported that the combination of graphene and PEDOT:PSS produces better sensing properties for working electrodes [19,20]. All fabrication steps were at room temperature and required no sophisticated printing techniques. Through the immobilization of aptamers, the newly conductive paper-based device demonstrated its sensitivity and selectivity of electrochemical measurements of CEA in serum samples. This inexpensive and disposable paper-based electrochemical sensor would provide a point-of-care biomarker analytical tool for cancer diagnostics in less industrialized or resource-limited areas where fabrication facilities and skilled personnel are limited.

## 2. Materials and Methods

### 2.1. Materials

DNA aptamer against CEA: 5’-NH2-GAC GAT AGC GGT GAC GGC ACA GAC GTC CCG CAT CCT CCG-3’ (Mw 12,143 g mol−1) [21] was synthesized and purified from AllBio Science Inc., Taiwan. CEA antigens (ab742) and Anti-CEA antibodies (ab131070) were purchased from Abcam. Graphene ink (I-MS18) was obtained from Enerage Inc., Taiwan. PEDOT:PSS 1.3 wt. %, phosphate-buffered saline (PBS) with pH 7.4, bovine serum albumin (BSA, A7030), prostate specific antigen (PSA, P3235), insulin (I9278), (3-Aminopropyl)triethoxysilane (APTES), succinic anhydride, human serum (H4522), K_3_[Fe(CN)_6_], KCl solution, methanol, ethanol and toluene solutions were all purchased from Sigma Aldrich, Taiwan. All reagents were of analytical grade and were used without any prior treatment. Whatman No. 3 qualitative filter paper and polydimethylsiloxane (PDMS) were bought from Yeong Jyi Chemical Apparatus Co., Taiwan. TWEEN 80 was obtained from First Chemical Manufacture Co., Taiwan. All aqueous solutions were prepared in ultra-pure Milli-Q water with a resistivity of 18.2 MΩ cm (at 25 °C).

### 2.2. Measurement and Apparatus

Electrochemical measurements were performed by a PalmSens4, equipped with PSTrace 5.6 software (PalmSens BV, Houten, The Netherlands). A three-electrode system was employed during electrochemical impedance spectroscopy (EIS) measurement. The platinum served as an auxiliary electrode and Ag/AgCl served as a reference electrode in 50 mM PBS containing 5 mM [Fe(CN)_6_]^3−/4−^ and 0.1 M KCl at a 0.1 V potential over the frequency range from 100 kHz to 1 Hz. A designed and integrated electrochemical cell (4 mL of sample volume) was used for all measurements, including a cover and reaction tank (Figures S1 and S2). A high torque DC brushless spin coater (SC-80R, YOTEC, Taipei, Taiwan) was used for coating the graphene ink onto the paper substrate. The conductivity of the graphene/PEDOT:PSS modified paper strips was measured using a source meter (Keithley 2400, Tektronix, Beaverton, OR, USA). Characteristic studies of the surface functionalization of the graphene/PEDOT:PSS paper electrode were performed using Fourier transform infrared (FTIR) analysis (Spotlight 200i Sp2, Perkin Elmer, Waltham, MA, USA). The surface morphology of the conductive paper electrode was characterized by a Scanning electron microscope (SEM, JSM-7610F, JEOL, Tokyo, Japan).

### 2.3. Fabrication of Graphene/PEDOT:PSS Modified Paper-Based Electrode

**Scheme 1:**
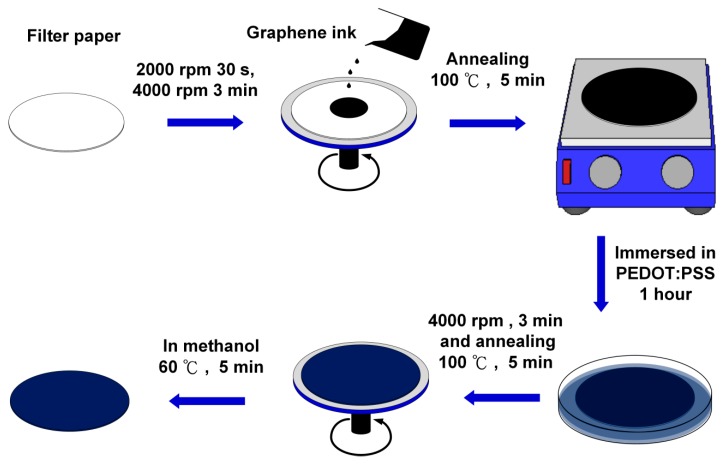
Fabrication and modification process of the electrochemical paper-based working electrode: (1) paper cleaning; (2) graphene ink spin coating and annealing; (3) poly (3,4-ethylenedioxythiophene):poly(styrenesulfonate) (PEDOT:PSS) dip coating; (4) PEDOT:PSS spin coating and annealing; and (5) methanol treatment on the paper electrode.

Scheme 1 shows the fabrication and modification process of the graphene/PEDOT:PSS paper-based sensing electrode. A Whatman No. 3 qualitative filter paper was preliminarily cleaned and decontaminated with deionized water containing 3 drops of Tween 80. Then, the paper was immersed in ethanol solution sonicating for 15 min. Next, the paper was rinsed with deionized water and kept in the oven at 60 °C for drying. After the cleaning process, the graphene ink was spin-coated on the filter paper at 4000 rpm for 3 min, and then, the paper was placed on a hotplate at 100 °C for 5 min for annealing. After coating the graphene ink, the filter paper was immersed in PEDOT:PSS solution for 1 hour, followed by spinning the filter paper at 4000 rpm for 3 min so that the PEDOT:PSS could be evenly distributed on the paper and annealed on a hotplate at 100 °C for 5 min. In order to enhance the conductivity of the graphene/PEDOT:PSS modified paper electrode, the paper was further treated with methanol at 100 °C for 5 min [22], then, dried in the air.

### 2.4. Surface Functionalization of the Conductive Paper-Based Aptasensor

**Figure 1:**
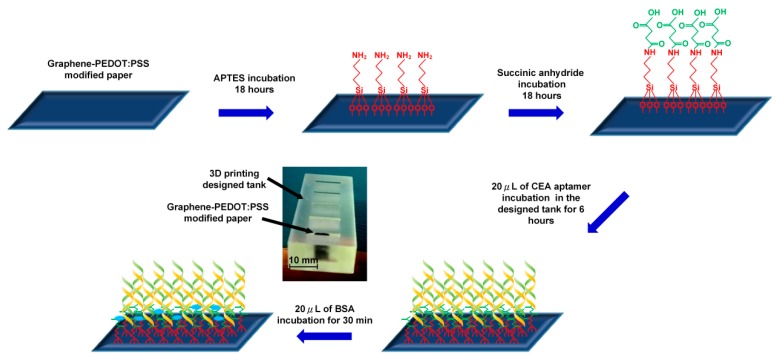
Surface functionalization process of the conductive paper-based electrochemical aptasensor.

Graphene/PEDOT:PSS modified paper was cut in a strip 10 mm in length and 4 mm in width for further functionalization processes as shown in Figure 1. The strip electrode was first incubated in a 1% (*v/v*) APTES solution (in toluene) for 18 hours to form an amine group on the electrode surface. After being rinsed with toluene, the strip electrode was then incubated in a 5% succinic anhydride (SA) solution (in toluene) for 18 hours to modify the electrode surface with a carboxyl function group. Next, the modified paper strip was immersed in a designed tank containing 20 μL of CEA aptamers (500 μg·mL^−1^) for 4 hours of incubation followed by washing with 1 mL of PBS to remove the unbound aptamers. Then, 10 μL of 1% BSA solution was employed to block any non-specific binding sites, and the paper electrode was washed with PBS. Finally, the prepared graphene/PEDOT:PSS modified paper-based aptasensor was stored at 4 °C for further use.

## 3. Results and Discussion

### 3.1. Surface Morphologies and Characterization

**Figure 2:**
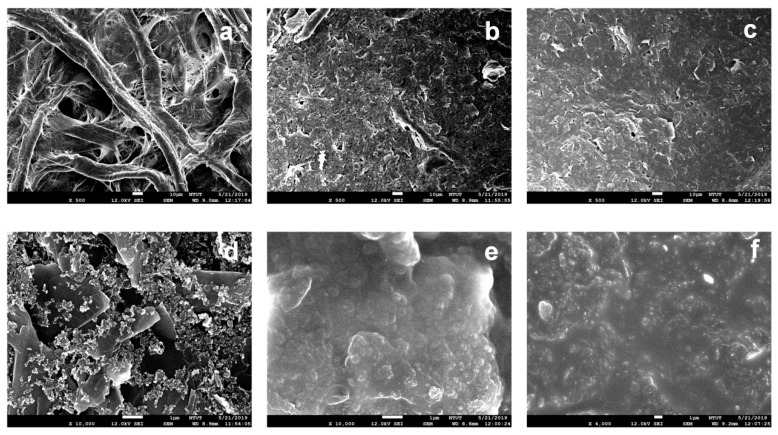
SEM images at 500× magnification of unmodified Whatman grade No. 3 filter paper (**a**) And filter paper modified with: (**b**) Graphene ink, (**c**) Graphene/PEDOT:PSS. SEM images at 10,000× magnification of the filter paper modified with: (**d**) Graphene ink, (**e**) Graphene/PEDOT:PSS, and (**f**) Graphene/PEDOT:PSS with methanol treatment.

Surface morphologies of each fabrication process of paper electrodes were further investigated through SEM analysis. Figure 2a shows the Whatman No. 3 filter paper not modified with other materials; the cellulose fiber distribution can be seen. Figure 2b,c shows SEM images (500× magnification) of the graphene-coated paper and graphene/PEDOT:PSS modified paper, respectively. It is observed that voids of fibers are roughly filled with graphene. With further absorption of PEDOT:PSS, the remaining interspace between the graphene and fibers as well as the interlayer spacing of graphene seem to be filled with PEDOT:PSS [23]. Figure 2d,e show the SEM images at 10,000× magnification of the paper electrode in three fabrication steps. Figure 2d shows the sheet structure of graphene, and Figure 2e clearly demonstrates that PEDOT:PSS is absorbed to fill the interspaces and flatten the surface morphology of the paper electrode. Figure 2f shows that the surface of the methanol treated graphene/PEDOT:PSS modified paper electrode is more homogenous than that of the untreated one. This improvement in the morphology enhances the electrical conductivity of the paper electrode [22].

**Figure 3:**
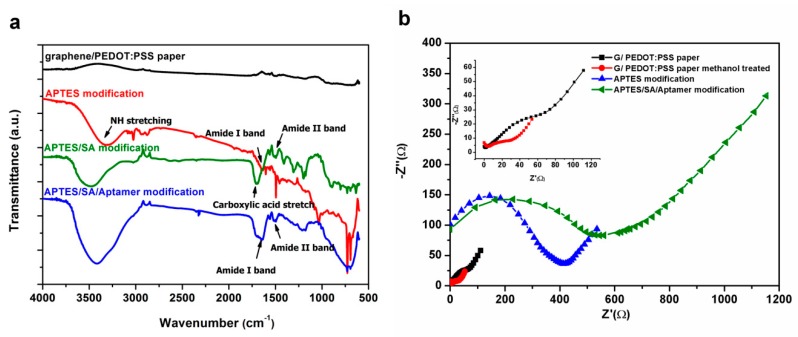
(**a**) The Fourier transform infrared (FTIR) spectra of a graphene/PEDOT:PSS paper electrode and (3-Aminopropyl)triethoxysilane (APTES) modified, APTES/SA modified and APTES/SA/Aptamer modified graphene/PEDOT:PSS paper electrode and (**b**) The electrochemical impedance spectroscopy (EIS) signal of graphene/PEDOT:PSS paper electrode in different surface functionalization steps.

To verify the surface functionalization of the aptasensor, FTIR was applied to characterize the paper electrode at each stage of modification. The FTIR spectra of the graphene/PEDOT:PSS paper electrode and each process of surface functionalization are shown in Figure 3a. In the APTES modification spectrum, the bending vibrations of NH_2_ stretching and –NH_2_ bending (amide I band) can be detected at 3350 cm^−1^ and 1605 cm^−1^ [24]. In the APTES/SA modification spectrum, peaks at 1555 cm^−1^ and 1700 cm^−1^ are assigned to the amide II band of secondary amide and carboxylic acid stretching after carboxylation [25]. In the final aptamer modification spectrum, peaks at 1650 cm-1 and 1555 cm-1 correspond to the amide I band and amide II band of the secondary amide. Moreover, the presence of peaks from the phosphate group stretching at 1215 cm^−1^ and 1035 cm^−1^ [26] may indicate the successful immobilization of aptamers on the graphene/PEDOT:PSS modified paper electrode.

In EIS measurement, the Randles circuit is used as an equivalent circuit for quantitative impedance analysis. Figure 3b shows the impedance analysis of the paper-based electrode in each stage of modification. In the representation of the Nyquist plot, the diameter of the semicircle gives the magnitude of the charge transfer resistance (R_ct_) at the electrode surface [27]. From the upper left corner of Figure 3b, it can be seen that the R_ct_ (19.64 Ω) of the graphene/PEDOT:PSS modified paper electrode impregnated with methanol (red circle) is smaller than that (36.04 Ω) of the unmodified methanol (black square) one, which confirms that alcohols can improve the electrical conductivity of PEDOT:PSS [22]. When the amino functional group of APTES (blue triangle) is modified on the surface of the paper electrode, it can block the function of partial electron transfer and increase the R_ct_ (358.4 Ω). From the plot of aptamer modification on the paper electrode, it can be seen that the R_ct_ significantly increases to 522.3 Ω which indicates that the macromolecule aptamers are well immobilized on the surface of the modified paper electrode.

### 3.2. Detection of CEA by the Paper-Based Aptasensor

**Figure 4:**
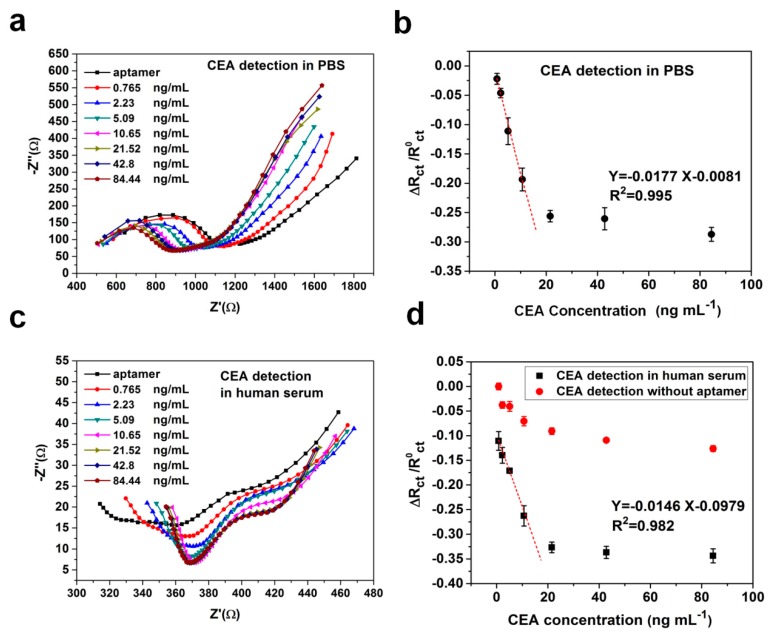
(**a**) Nyquist plot of EIS responses to different concentrations of carcinoembryonic antigens (CEA) in 0.1 M PBS solution (pH = 7.4); (**b**) The calibration curve between the relative change rate of R_ct_ and concentration of CEA in PBS samples (*n* = 5); (**c**) Nyquist plot of EIS responses to different concentrations of CEA in human serum samples; (**d**) The calibration curve between the relative change rate of R_ct_ and concentration of CEA in human serum samples (*n* = 5).

The sensitivity and dynamic range of CEA aptamer sensors were examined by measuring the change in charge transfer resistance (R_ct_) of the equivalent circuit model from the EIS impedance spectrum. Each paper-based aptamer sensor was tested in a single biological sample containing a concentration of CEA in a range from 0.77 to 84.44 ng·mL^−1^. The number of test samples of each concentration is five (*n* = 5). The detection of analytes is based on the extent to which the R_ct_ varies due to the formation of an aptamer-antigen complex on the paper electrode’s surface. In addition, aptasensors were also tested both in the standard PBS and human serum samples. The measurements of both samples, the Nyquist plots of EIS responses, are shown in Figure 4a,c. It was observed that with the increase in CEA concentration, the relative charge transfer resistances decreased gradually. Figure 4b,d shows calibration plots illustrating good linear detection relationships between the relative change rate of R_ct_ and the concentration of CEA in the ranges of 0.77–14 for PBS samples (*R*^2^ = 0.995) and for human serum samples (*R*^2^ = 0.982), respectively. Signals of paper-based aptasensors were found to be saturated at the CEA concentration above 21 ng·mL^−1^, which could be improved by adding nanomaterials to increase the sensing surface area and electrical connectivity [28]. However, the additional nanomaterials could also raise the cost of the sensor. In addition, conductive paper-based sensors without modifying CEA aptamers were employed for CEA detection in human serum samples, and significantly weak responses are shown in Figure 4d. The regression equation obtained between the relative change rate of R_ct_ and the concentration of CEA was ΔR_ct_\R^0^_ct_ = −0.0177 [C_CEA_] (ng·mL^−1^) - 0.0081 for PBS samples and ΔR_ct_\R^0^_ct_ = −0.0146 [C_CEA_] (ng·mL^−1^) - 0.0979 for human serum samples, respectively. The limit of detection (LOD) was defined as the concentration that can be detected at three times the standard deviation σ of the blank signal. This was calculated to be 0.45 ng·mL^−1^ for PBS samples and 1.06 ng·mL^−1^ for human serum samples, separately. The results demonstrated that the graphene/PEDOT:PSS modified paper-based aptasensor had a capability to be applied for typically clinical practice in detecting CEA in which its cut-off value was 5 ng·mL^−1^ for the diagnosis of cancer.

### 3.3. Repeatability and Selectivity of the Conductive Paper-Based Aptasensor

**Figure 5:**
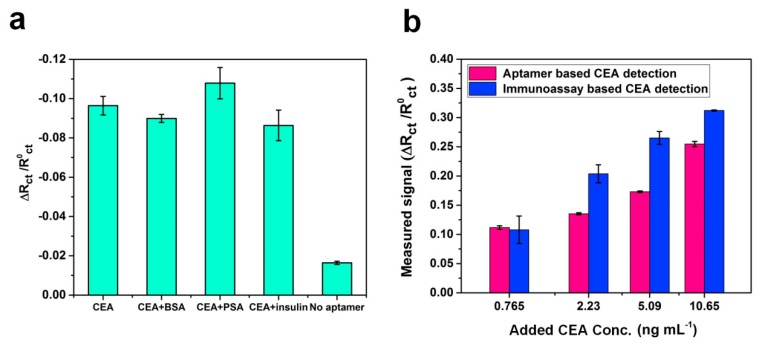
(**a**) Comparison of R_ct_ response of aptasensors in the presence of other analytes including bovine serum albumin (BSA), prostate specific antigen (PSA), and insulin, as well as the working electrode without immobilizing CEA aptamers; (**b**) Comparison of R_ct_ response of the proposed paper-based aptasensor and paper-based immunoassay sensing method.

Repeatability of a disposable paper-based aptasensor was examined in both PBS and serum samples. Five different modified paper electrodes were scanned in samples by PalmSens4. As shown in Figure S3a,b, we evaluated the R_ct_ for a graphene/PEDOT:PSS/CEA aptamer modified paper electrode and found the coefficient of variation was 0.26% (*n* = 5) for an electrode in the PBS solution and 0.64% (*n* = 5) in the serum. The results show that this low-cost graphene/PEDOT:PSS/aptamer modified paper electrode has good repeatability for sensing. In order to verify the selectivity of the paper-based aptasensor, 5 ng·mL^−1^ of CEA was measured in the presence of 10 ng·mL^−1^ of interfering molecules, including bovine serum albumin (BSA), prostatic specific antigen (PSA) and insulin. As shown in Figure 5a, the variation in relative change rate of R_ct_ was less than 10% on addition of other molecules. The paper electrode without CEA aptamers immobilization exhibited a weak response due to the nonspecific adsorption. All results indicate that the proposed aptasensor has good specificity for CEA.

### 3.4. Serum Sample Analysis

In order to test the applicability of the system, a graphene/PEDOT:PSS modified paper-based aptasensor was used to detect CEA in human serum, and the results were compared with a CEA immunoassay sensor. The blank human serum samples were applied for the determination of accuracy and validation of a new clinical sensing assay. Different concentrations of CEA (0.765, 2.23, 5.09, and 0.65 ng·mL^−1^) were spiked into the human serum, and samples were detected directly by the sensor without any dilution. The results indicate that R_ct_ responses depend on CEA concentrations. As expressed in Table 1, the percentages of recoveries were found to be in the range of 98.1%–118.5%, and the RSD was less than 5.0% except for the sample with the CEA concentration of 0.765 ng·mL^−1^. In Figure 5b, it is observed that a reasonable correlation occurs between the CEA concentration value obtained by using the immunoassay method and the graphene/PEDOT:PSS modified paper-based aptasensor. The comparison between this work and other CEA sensing methods reported in literature is summarized in Table 2. It was found that works developing paper-based CEA sensors all claimed a low-cost advantage compared to the commercial ECLIA method. Moreover, synthetic nanomaterials were applied in several works to improve the sensitivity of the aptasensors [15,29,30], but these may also increase the cost. In addition, compare with the paper-based sensor with EIS detection for CEA [14], the electrode developed in this study was modified by graphene together with PEDOT:PSS, which improves the sensitivity of the sensor. Furthermore, the CEA aptamer modification was applied instead of using antibody immobilization for the paper-based sensor can greatly reduce the developing expense of the device. In brief, from the comparison of listed features such as electrode substrate material, fabrication method, detection range and cost, the sensor developed in this work has the following benefits: (1) The fabrication method of the working electrode is simple and cost-effective. All processes are at room temperature, are made on a paper substrate and require no complicated and costly coating instruments; (2) The conductive paper-based working electrode with aptamer surface immobilization without adding other synthetic nanomaterials or nanoparticles for signal amplification, which simplifies the process and reduced costs. The cost of a paper-based aptasensor was roughly estimated to be $ 0.3; (3) the special electrochemical cell (4 mL of sample volume) was developed for the portable sensing platform. Therefore, the proposed sensor may achieve to be a portable and disposable POC device with a reasonable sensitivity for the medical demand in a resource-limited area.

## 4. Conclusions

We have developed an electrochemical paper-based aptasensor for CEA detection, based on a straightforward and continuous graphene/PEDOT:PSS modification process. The conductive paper electrode was further chemically functionalized in a specially designed reactor for the immobilization of CEA aptamer. The proposed aptasensor was applied for sensing CEA with EIS analysis. These results displayed a good linearity in the range of 0.76–14 ng·mL^−1^ and a low limit of detection for CEA. In addition, this sensing system was successfully applied for the determination of CEA in human serum and compared with the immunoassay method. This method does not require complicated fabrication techniques, costly substrates, or nanomaterials demanding specialized synthetic techniques. This new and sensitive paper-based aptasensor might be an alternative POC tool for cancer markers screening in less developed countries or resource limited areas.

## Figures and Tables

**Table 1:** Determination of CEA in human serum samples (**n** = 3).

CEA Concentration (ng·mL^−1^)		% Recovery
Added	Measured	
0.765	0.91 ± 0.21	118.5
2.23	2.49 ± 0.11	111.5
5.09	5.00 ± 0.10	98.2
10.65	10.44 ± 0.30	98.1

**Table 2:** Comparisons of reported biosensors and their sensing characteristics towards CEA.

No.	Detection Method	Electrode	Fabrication Method	Linear Range (ng·mL^−1^)	LOD (ng·mL^−1^)	Cost	Reference
1	Commercial ECLIA	Ion selective electrode	-	0.2–1000	0.2	Very High	[10]
2	SAW with immunoassay	LiTaO_3_ substrate	Micromachining	1–12	1.25	High	[11]
3	Amperometry with immunoassay	PEDOT:PSS/RGO modified Whatman paper 1	Dip coating	2–8	-	Low	[31]
4	ECL with aptamer	glassy carbon electrode	Surface modification	0.01–10.0	0.0038	Moderate	[29]
5	DPV with aptamer	Whatman paper 1; gold electrode	Wax printing and screen-printing; surface modification	0.01–500; 5–40	0.002; 3.4	Low	[15,30]
6	UC- FRET with aptamer	Employing upconversion phosphors	Chemical synthesis	0.1–40	0.1	high	[32]
7	EIS with immunoassay	PEDOT:PSS modified Whatman paper No. 1	Dip coating	1–25	2.68	Low	[14]
8	EIS with aptamer	Graphene-PEDOT:PSS modified Whatman paper No. 3	Spinning coating and dip coating	0.77–14	0.45	Low	This work

Surface acoustic wave (SAW), electrochemiluminescence (ECL), electrochemical impedance spectroscopy (EIS), differential pulse voltammetry (DPV), and upconversion fluorescence resonance energy transfer (UC-FRET).

## Supplementary Materials

The following are available online at https://www.mdpi.com/1424-8220/20/5/1372/s1, Figure S1: The design plot of the 3D printed cover for electrochemical cell and the picture of the real printed product, Figure S2: The picture of the specially designed and integrated electrochemical cell for the graphene/PEDOT:PSS modified paper-based aptasensor, Figure S3: Five different modified paper electrodes were scanned in (a) 0.1M PBS solution and (b) human serum sample by electrochemical workstation, separately.
