# Supplementary material for: A Graphene-PEDOT:PSS Modified Paper-Based Aptasensor for Electrochemical Impedance Spectroscopy Detection of Tumor Marker
Source: Sensors (Basel). 2020 Mar 2;20(5):1372. doi: 10.3390/s20051372 (PMC7085636; doi:10.3390/s20051372)
Supplement: Supplementary file 1 [file sensors-20-01372-s001.pdf]

sensors

A graphene-PEDOT:PSS Modified Paper-Based Aptasensor for Electrochemical Impedance Spectroscopy Detection of Tumor Marker

Yi-Kuang Yen[a,*], Chen-Hsiang Chao[b] and Ya-Shin Yeh[a]

[a] Institute of Mechatronic Engineering, National Taipei University of Technology, Taipei 106, Taiwan
[b] Taiwan Semiconductor Manufacturing Company, Hsinchu 30078, Taiwan
* Corresponding: Yi-Kuang Yen (ykyen@ntut.edu.tw)

Experimental

Design, fabrication and integration of the electrochemical reaction cell

Firstly, we used a stereolithography (SLA) 3D printer to make a cover for fixing electrodes of the electrochemical cell. The advantage of selecting 3D printing is that it can be made into any size and structure, so that the diameter of the electrode port on the cover is just tightly matched with the electrode, and it can be compatible with any container. The design of the cover was shown as Figure S1. Three surrounding ports were for the electrodes and the central port was for the sample injection.

In the part of the electrochemical reaction cell, we integrated a 25 mL beaker with a polydimethylsiloxane (PDMS) which has high biocompatibility and inertness, as shown in Figure S2. This method can reduce the volume of reaction cell to 4 mL, which not only can miniaturize the electrochemical sensing system, but also the length of the working electrode can be shortened, and greatly decrease the cost of surface modification of the electrode.

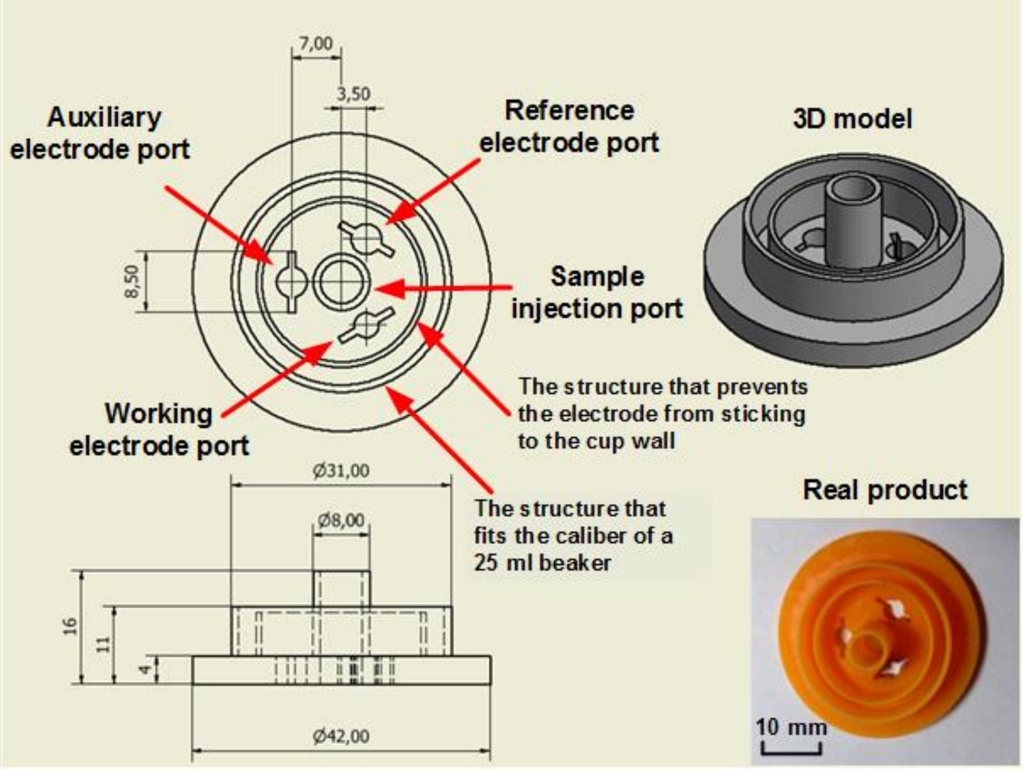

Figure 1. The design plot of the 3D printed cover for electrochemical cell and the picture of the real printed product.

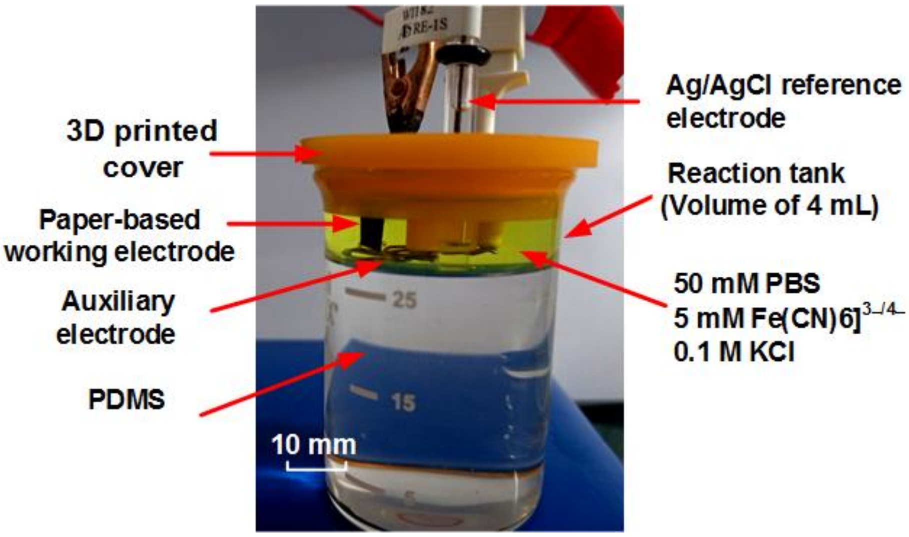

Figure 2. The picture of the specially designed and integrated electrochemical cell for the graphene/PEDOT:PSS modified paper-based aptasensor.

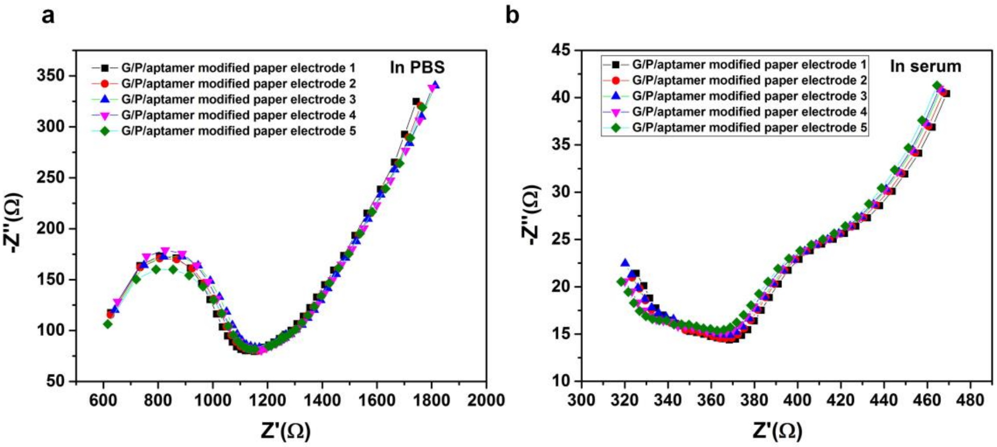

Figure 3. Five different modified paper electrodes were scanned in (a) 0.1M PBS solution and (b) human serum sample by electrochemical workstation, separately.
